# Regulation of TMEM2-mediated hyaluronan degradation by CD44 and LYVE-1

**DOI:** 10.3389/fmolb.2026.1852012

**Published:** 2026-08-12

**Authors:** Yuki Tobisawa, Fumiakira Yano, Risa Tomioka-Inagawa, Fumitoshi Irie, Takuya Koie, Yu Yamaguchi

**Affiliations:** 1 Department of Urology, Gifu University Hospital, Gifu, Japan; 2 Center for One Medicine Innovative Translational Research (COMIT), Institute for Advanced Study, Gifu University, Gifu, Japan; 3 Center for Neurologic Diseases, Sanford Burnham Prebys Medical Discovery Institute, La Jolla, CA, United States

**Keywords:** CD44, glycocalyx, hyaluronan, hyaluronidase, LYVE-1, TMEM2

## Abstract

Hyaluronan (HA) is a major glycosaminoglycan of the extracellular matrix that regulates cell migration, signaling, and tissue homeostasis. Its turnover is controlled by coordinated synthesis by HA synthases and degradation by hyaluronidases. Among these hyaluronidases, TMEM2, the only known transmembrane hyaluronidase, plays a unique role in HA degradation at the cell surface; however, the cellular conditions that support its activity remain incompletely understood. To address this, we developed a cell-based HA turnover assay to examine TMEM2-mediated degradation of endogenously synthesized HA, rather than exogenously added, fluorescently labeled HA used in previous studies. Using this system, we show that TMEM2 readily degrades high-molecular weight HA synthesized by co-expressed HAS3. This degradation occurs only when TMEM2 and HAS3 are co-expressed in the same cells (*cis*), whereas co-culture of TMEM2-expressing cells with HAS3-expressing cells supports little or no degradation. Interestingly, HA-binding cell surface receptors CD44 and its homolog LYVE-1 promote efficient TMEM2-mediated HA degradation even under *trans* conditions, whereas other HA-binding proteins, including TSG-6, layilin, TLR2, RHAMM, and ICAM-1, do not. These findings suggest a spatially regulated mechanism of TMEM2 activity in which capture of HA at the cell surface, mediated by CD44 or LYVE-1, contributes to efficient HA degradation by TMEM2.

## 1 Introduction

Hyaluronan (HA) is a large glycosaminoglycan that is widely distributed throughout the body as a major component of the extracellular matrix. It consists of a linear polymer of repeating disaccharide units of N-acetylglucosamine and glucuronic acid. HA is synthesized at the plasma membrane by hyaluronan synthases (HAS1, HAS2, and HAS3), which directly extrude the growing polymer into the extracellular space. HA undergoes remarkably rapid biological turnover in tissues, with approximately one-third of the total body HA pool being replaced daily (Fraser and Laurent, 1989).

Cellular catabolism of HA is mediated by an array of hyaluronidases that act in distinct cellular compartments or physiological contexts. These include the HYAL family hyaluronidases (HYAL1, HYAL2, and SPAM1), HYBID (also known as CEMIP or KIAA1199), and TMEM2 (Lepperdinger et al., 1998; Stern and Jedrzejas, 2006; Triggs-Raine et al., 1999; Yamaguchi, 2025; Yoshida et al., 2013). Among these proteins, TMEM2 is unique as a transmembrane hyaluronidase that degrades HA at the cell surface (Yamamoto et al., 2017). Recent studies indicate that TMEM2 plays a critical functional role at both cellular and organismal levels. Studies of knockout mice lacking this enzyme revealed that TMEM2 is crucial for HA metabolism in adult animals (Tobisawa et al., 2021). During mouse development, *Tmem2*-mediated HA degradation is essential for neural crest cell migration and enamel formation in teeth (Inubushi et al., 2022; Nag et al., 2023). These findings highlight the physiological importance of TMEM2 *in vivo*.

While the enzymatic properties of TMEM2 have been characterized (Narita et al., 2023; Yamamoto et al., 2017), the cellular mechanisms by which TMEM2 degrades HA at the cell surface remain poorly understood. In particular, the relatively slow catalytic activity of human TMEM2 (Narita et al., 2023), contrasted with the striking metabolic and developmental phenotypes observed in *Tmem2* ^*−/−*^ mice (Inubushi et al., 2022; Tobisawa et al., 2021), suggests the involvement of accessory molecules that facilitate its full enzymatic activity in cellular and physiological contexts. One potential mechanism is that certain HA-binding proteins participate in TMEM2-mediated HA degradation at the cell surface by confining HA in close proximity to TMEM2.

In this study, we investigated the cellular conditions that enable human TMEM2 to degrade endogenously synthesized, native HA. We show that TMEM2 readily degrades HA when TMEM2 is co-expressed with HAS3 in the same cells. Moreover, co-expression of link module-containing transmembrane receptors, namely, CD44 and LYVE-1, but not other types of HA-binding proteins, significantly enhances TMEM2-mediated HA degradation. These findings suggest that cooperation with these link module-containing cell surface HA receptors is critical for enabling TMEM2 to achieve robust HA degradation at the cell surface.

## 2 Materials and methods

### 2.1 Expression vectors

For expression of human TMEM2, TSG-6, and TLR2, the following premade expression vectors were purchased and used directly to express respective proteins: pFN21a-TMEM2-Halo (Promega), pCMV-SPORT6-TNFAIP6 and pCMV-SPORT6-TLR2 (DNAFORM, Yokohama, Japan). Human HAS3 cDNA was a gift from Dr. Mark Tammi (University of Eastern Finland, Kuopio, Finland) and was subcloned into pcDNA3 (Invitrogen). For expression of human CD44 (CD44v, 699 amino acids) and LYVE-1, cDNAs encoding the respective proteins were amplified from pGEM-hCD44 (Sino Biological, HG12211-G) and pMD-hLYVE1 (Sino Biological, HG10237-M), respectively, and subcloned into pcDNA3.1/myc-HisA (Invitrogen). For expression of human ICAM-1 and CD62L, cDNAs encoding the respective proteins were amplified from pOTB7-ICAM1 (DNAFORM, 3506766) and pDNR-LIB-SELL (DNAFORM, 4717456), respectively, and subcloned into pcDNA3.1. cDNAs encoding human layilin and RHAMM were amplified from pBluescriptR-LAYN and pCR-BluntII-TOPO-HMMR (DNAFORM, Yokohama, Japan), respectively, engineered to include a C-terminal FLAG epitope (DYKDDDDK) by PCR, and subcloned into pcDNA3.1.

### 2.2 Cells

293 cells (CRL-1573), a human embryonic kidney cell line, and 293T cells (CRL-3216), a highly transfectable 293 derivative, were acquired from the American Type Culture Collection (Manassas, VA). These cells were selected for their low endogenous HA production and high suitability for transfection-based assays. Cells were cultured in Dulbecco’s modified essential medium (DMEM; FUJIFILM Wako Pure Chemical Corporation) supplemented with 100 U/mL penicillin, 100 μg/mL streptomycin, and 10% fetal bovine serum. For transient expression, 293T cells were transfected using TransIT-293 (Mirus Bio, USA), as described below. To establish TMEM2 stable cell line (293^TMEM2^), 293 cells were transfected with pFN21a-TMEM2-Halo and pcDNA3.1 in a 10:1 ratio. Transfectant were selected in the presence of G418.

### 2.3 Hyaluronan degradation assays

HA degradation assays with transfected cells were performed essentially as described previously (Yamamoto et al., 2017). 293T cells in 12-well plates (6 × 10^5^ cells per well) were transfected with expression vectors encoding human HAS3, TMEM2, and/or CD44, using TransIT-293 (Mirus Bio, USA). Controls were transfected with insertless pcDNA3.1. After 20 h, transfected cells were reseeded into 24-well plates (4 × 10^5^ cells per well), and culture supernatants were collected after 48 h of incubation. HA degradation was analyzed by size-exclusion chromatography on a 0.5 × 13 cm Sepharose CL-2B (Millipore) column calibrated with three fluoresceinamine-conjugated HA standards (FAHA-H2, 1,200–1,600 kDa, lot 18H487; FAHA-L2, 100–300 kDa, lot 18J499; and FAHA-U2, 5–10 kDa, lot 17J447). Culture supernatant samples (0.1 mL) were applied to the column equilibrated with PBS and run at a flow rate of 0.17 mL/min. Fractions were collected at 0.2 mL per fraction. HA concentration in each fraction was quantified using the Hyaluronic Acid LT Assay (Fujifilm Wako Chemicals, lower limit of quantification, 10 ng/mL). For quantitative analysis of HA degradation, the cumulative HA signal eluted in fractions 5–8, which correspond to the elution positions of the high-molecular-weight HA standard (FAHA-H2), was taken to represent largely intact or minimally degraded HA species and was measured for each experimental condition. HA degradation activity was calculated as the percentage reduction in this signal relative to that in the untreated control. Statistical analyses were performed using one-way ANOVA followed by the *post hoc* test specified in each figure legend. Data are presented as mean ± SD from three independent experiments, each using separately transfected cells that were analyzed independently. P values < 0.05 were considered statistically significant.

### 2.4 Flow cytometry

293^TMEM2^ cells in 12-well plates (6 × 10^5^ cells/well) were transiently transfected with expression vectors encoding human CD44, ICAM-1, TLR2, or CD62L using TransIT-293 (Mirus Bio, USA). After 24 h, the cells were incubated with or without fluorescence-conjugated primary antibodies in 100 μL of 1% BSA–PBS for 30 min on ice. Flow cytometry was performed using the following antibodies: Alexa Fluor 647 anti-mouse/human CD282 (TLR2) recombinant antibody (1:100, Biolegend 153007), PE (phycoerythrin)-conjugated anti-mouse/human CD44 (1:100, Biolegend 103007), PE-conjugated anti-human CD62L (1:100, Biolegend 304805). For detection of ICAM-1, anti-human CD54 (ICAM-1) (Biolegend 353101) and PE-conjugated F(ab’)2-goat anti-mouse IgG (H+L) (1:200, Invitrogen 12-4010-82) were used. Cytometric data were acquired on CytoFLEX S (Beckman Coulter).

### 2.5 Immunoblotting

Total cell lysates were prepared using 1 × RIPA buffer with a protease inhibitor cocktail (Roche), separated on a 4%–15% gradient gel (Bio-Rad), and transferred onto a polyvinylidene difluoride membrane. Membranes were probed with the following primary antibodies: anti-FLAG tag antibody (1:1,000, GenScript A00187) to detect layilin and RHAMM; anti-LYVE-1 antibody (1:1,000, abcam ab219556, clone EPR21857); anti-TSG-6 antibody (1:1,000, Abcam ab267469, clone EPR17582-45); and anti-β-actin antibody (1:2000, Cell Signaling Technology 4970S, clone 13E5). The following secondary antibodies were used: HRP (horseradish peroxidase)–conjugated anti-rabbit IgG (1:2,000, Cell Signaling Technology 7074S) and HRP-conjugated anti-mouse IgG (1:2,000, Cell Signaling Technology 7076S). Immunoreactive signals were detected using SuperSignal West Pico chemiluminescent substrate (Thermo Fisher Scientific) and imaged with an LAS-4000 system (Fujifilm Wako Chemicals).

## 3 Results

### 3.1 TMEM2-mediated degradation of endogenously synthesized HA

**FIGURE 1:**
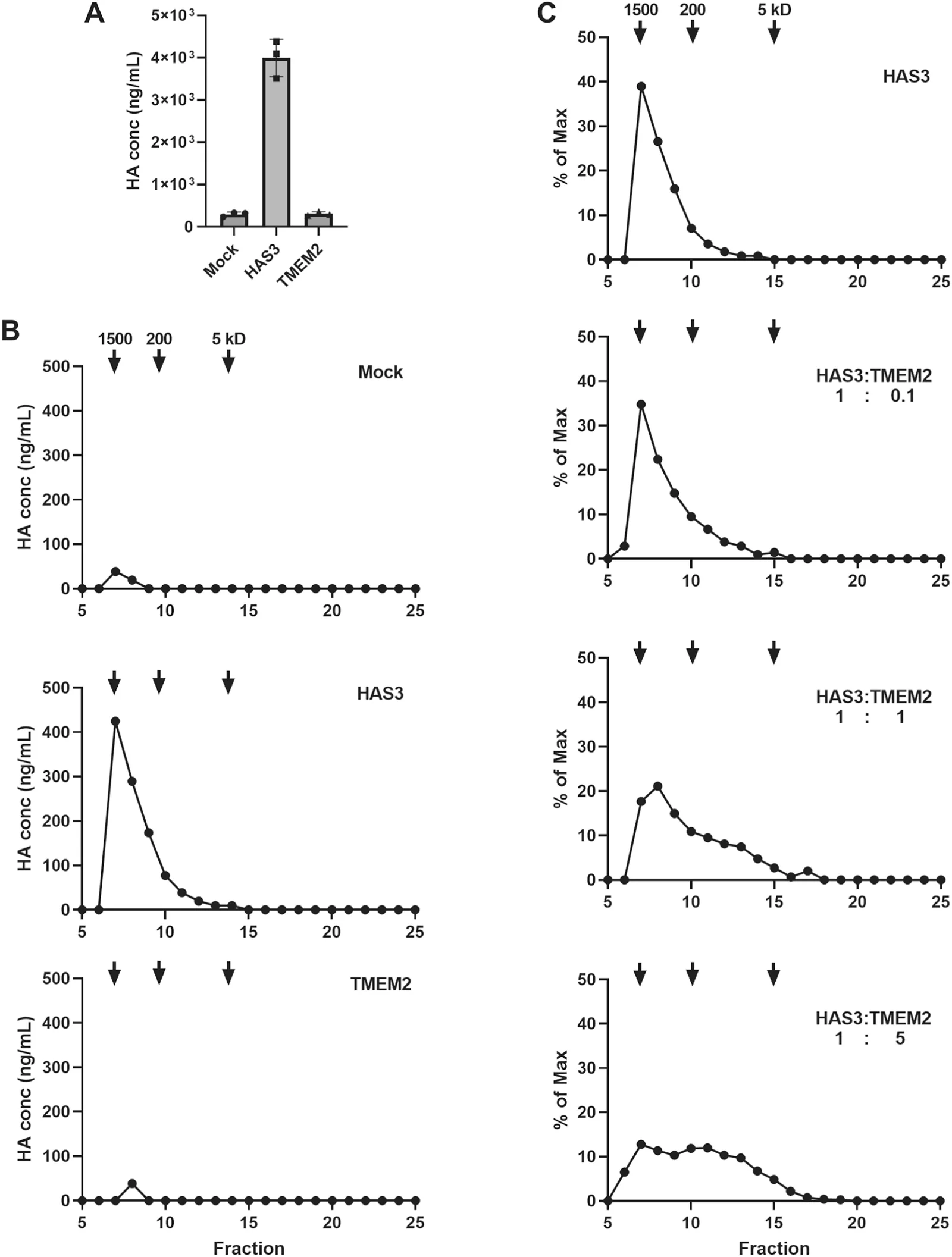
TMEM2 degrades endogenously synthesized HA in a cellular context. **(A,B)** HA production by 293T cells transfected with HAS3. Mock-transfected 293T cells and 293T cells transfected with HAS3 or TMEM2 were culture for 4 days, and HA production and the molecular weight distribution of HA were analyzed by HA quantification **(A)** and size-exclusion chromatography **(B)**, respectively. Data in the bar graph are presented as the mean ± SD from three independent experiments. **(C)** Co-transfection of HAS3 and TMEM2 at varying ratios results in progressively increased HA degradation with increasing amounts of TMEM2.

TMEM2-mediated HA degradation has mostly been studied using fluorescently labeled HA as a readily assayable exogenous substrate (Yamamoto et al., 2017; Sato et al., 2023). To examine the effects of TMEM2 on endogenously synthesized HA, rather than on exogenously added, chemically labeled HA, we established a cell-based assay using 293T cells. Mock-transfected 293T cells produce negligible amounts of HA in the absence of hyaluronan synthase transfection (Figure 1A). Transfection of HAS3 results in robust production of endogenously synthesized HA. Size-exclusion chromatography showed that HA species produced by HAS3-transfected cells were of high-molecular weight, with a peak size of ∼1,500 kDa (Figure 1B). In contrast, transfection of TMEM2 had no effect on HA production or size distribution (Figures 1A,B).

To evaluate the effect of TMEM2 co-expression on the degradation of endogenous HA, cells were co-transfected with HAS3 and TMEM2 at plasmid input ratios of 1:0.1, 1:1, and 1:5, with the total amount of HAS3 plasmid held constant (0.1 µg). Increasing the relative amount of TMEM2 expression plasmid resulted in progressively greater HA degradation (Figure 1C), indicating that TMEM2 can act on endogenously synthesized HA. Although this did not result in the complete depolymerization into ∼5–10 kDa fragments, the smallest size on which purified human TMEM2 can act (Narita et al., 2023), this partial degradation provides a useful assay system for evaluating the promoting effects of different conditions on TMEM2-mediated HA degradation.

### 3.2 Comparison of TMEM2-mediated HA degradation under *cis* and *trans* conditions

**FIGURE 2:**
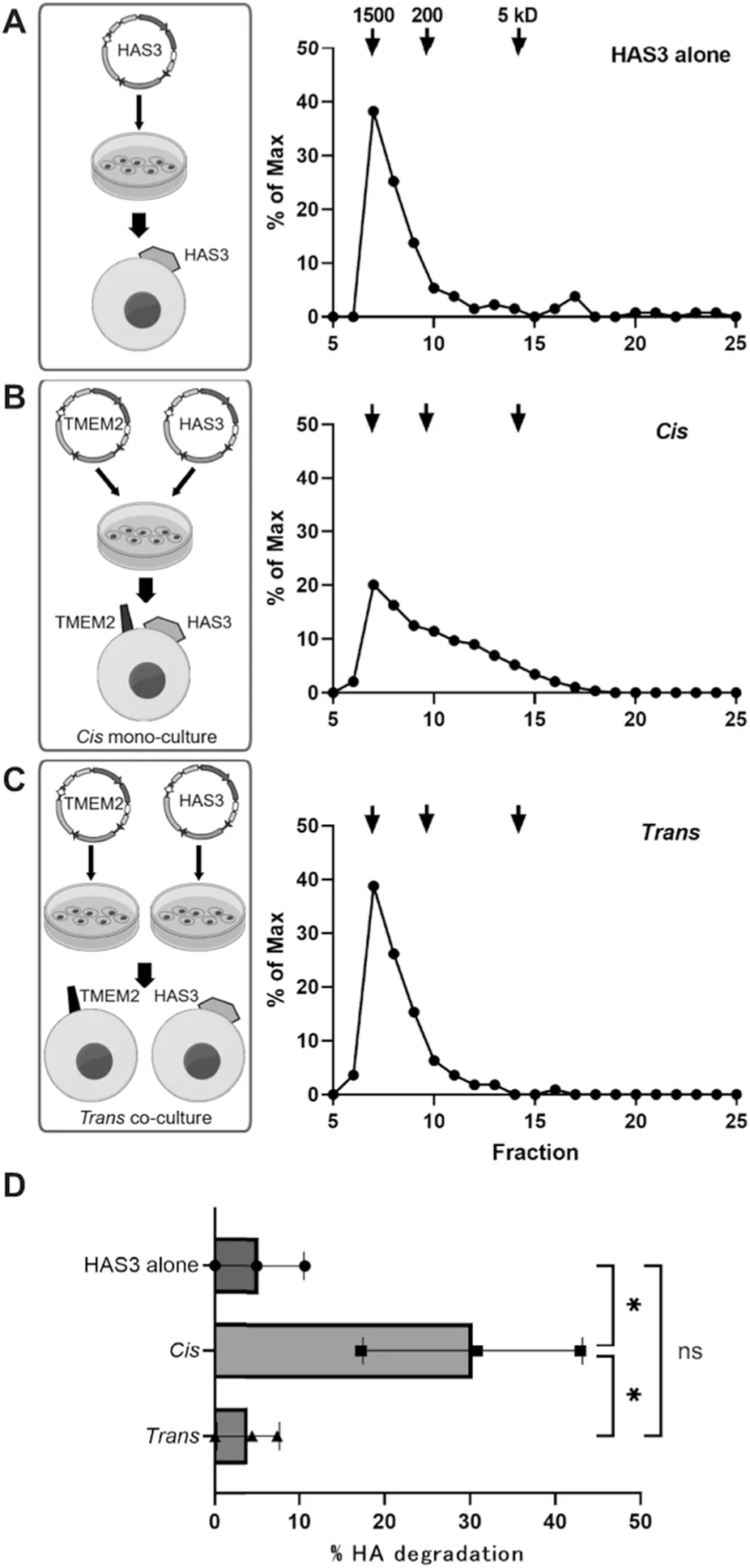
Comparison of TMEM2-mediated HA degradation under *cis* and *trans* conditions. **(A)** Control: 293T cells were transfected with HAS3 alone. **(B)** *Cis* condition: 293T cells co-transfected with HAS3 and TMEM2. **(C)** *Trans* condition: HAS3-transfected and TMEM2-transfected 293T cells were mixed at a 1:1 ratio and co-cultured. HA size distribution in culture supernatants was analyzed by size-exclusion chromatography. **(D)** HA degradation activity was calculated as described in *Materials and Methods*. Data are presented as mean ± SD from three independent experiments. Statistical significance was determined using one-way ANOVA followed by Tukey’s multiple comparisons test. *P < 0.05; *ns*, not significant.

The above results demonstrate that TMEM2 can degrade HA produced in the same cells (*cis* condition). We next asked whether TMEM2 degrade HA produced in separate cells (*trans* condition). To address this question, we compared HA degradation under *cis* and *trans* conditions (Figures 2A–C). In the *trans* condition, HAS3-transfected and TMEM2-transfected cells were mixed at a 1:1 ratio and co-cultured. This experiment showed that, whereas substantial HA degradation was detected under the *cis* condition, little or no degradation was detected under the *trans* condition (see Figure 2D for quantification). These results suggest that spatial relationship between TMEM2 and its HA substrate influences efficient HA degradation by TMEM2.

### 3.3 CD44 facilitates TMEM2-mediated HA degradation

We previously showed that TMEM2 degrades HA immobilized on glass slides (Irie et al., 2021; Yamamoto et al., 2017), indicating that *trans* degradation by TMEM2 is possible when HA mobility is constrained. This suggests that the inefficient HA degradation observed in the cell-based *trans* assay, shown in Figure 2, may result from the greater mobility of newly synthesized HA at the cell surface. We therefore hypothesized that efficient TMEM2-mediated HA degradation requires the presence of HA-binding proteins that restrict the mobility of HA.

**FIGURE 3:**
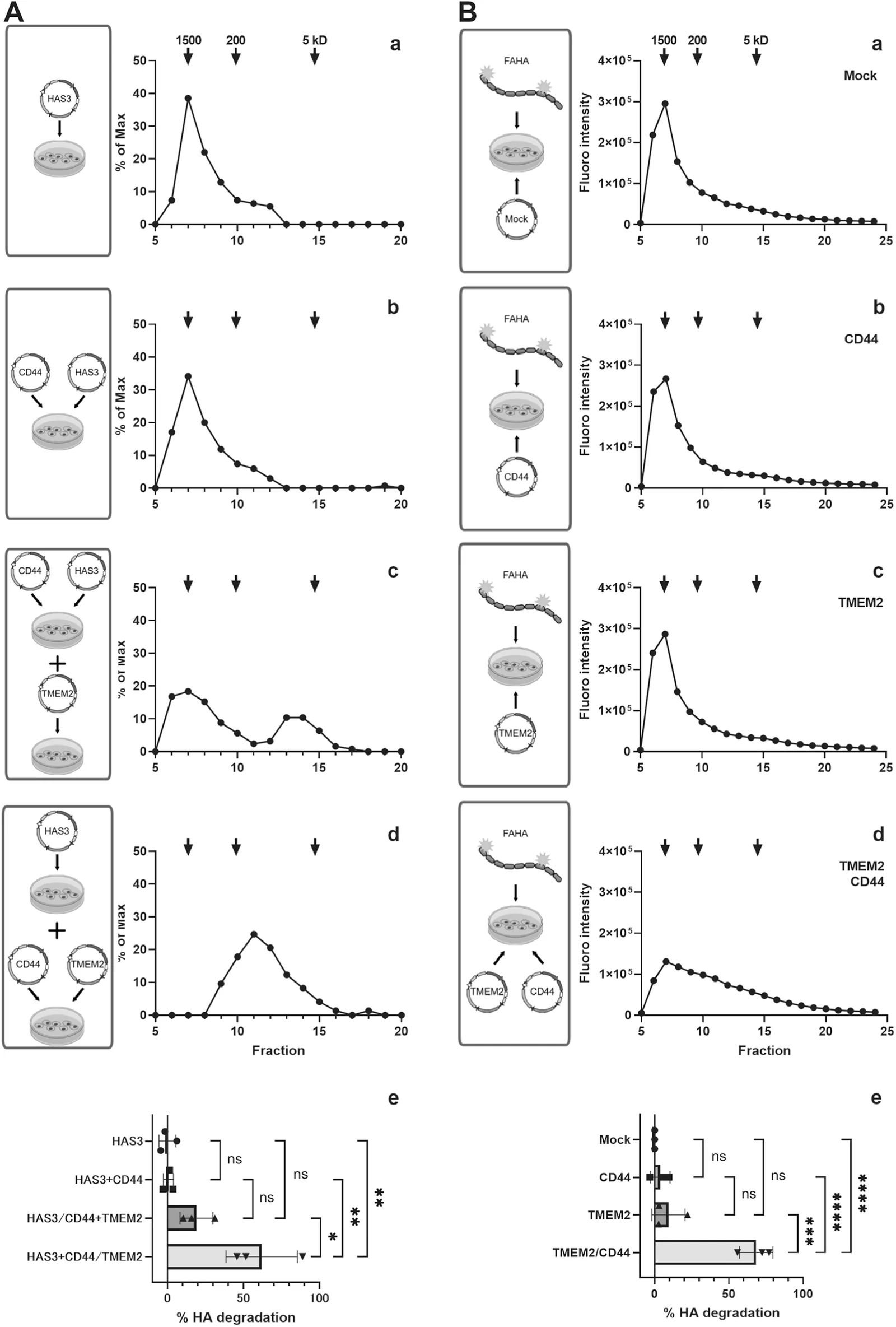
Co-expression of CD44 facilitates TMEM2-mediated HA degradation. **(A)** Effects of CD44 on TMEM2-mediated HA degradation under *trans* co-culture conditions. The following 293T transfectants were analyzed for their ability to degrade HAS3-synthesized HA under these *trans* conditions: (a) 293T cells transfected with HAS3 alone. (b) 293T cells co-transfected with HAS3 and CD44. (c) 293T cells co-transfected with HAS3 and CD44 were co-cultured with TMEM2-transfected cells. (d) 293T cells transfected with HAS3 were cocultured with cells co-transfected with CD44 and TMEM2. (e) Quantitation of HA degradation activity. Data are presented as mean ± SD from three independent experiments. Statistical significance was determined using one-way ANOVA followed by Tukey’s multiple comparisons test. *P < 0.05; **P < 0.01; *ns*, not significant. **(B)** Effects of CD44 on TMEM2-mediated degradation of diffusible HA. FA-HA was incubated with the following transfected 293T cells: (a) Mock-transfected 293T cells. (b) 293T cells transfected with CD44 alone. (c) 293T cells transfected with TMEM2 alone. (d) 293T cells co-transfected with CD44 and TMEM2. (e) Quantitation of HA degradation activity. Data are presented as mean ± SD from three independent experiments. Statistical significance was determined using one-way ANOVA followed by Tukey’s multiple comparisons test. *P < 0.05; ***P < 0.001; ****P < 0.0001; *ns*, not significant.

To test this hypothesis, we examined whether expression of the prototypical cell surface HA receptor CD44 promotes TMEM2-mediated HA degradation in *trans* assays. In the first experiment, CD44 was co-transfected with HAS3 in 293T cells, which were then cocultured with TMEM2-transfected cells (Figure 3A). While *trans* cocultures without CD44 showed little HA degradation, co-expression of CD44 induced partial yet substantial HA degradation (Figure 3A-c). In the second experiment, 293T cells were co-transfected with CD44 and TMEM2 and cocultured with HAS3-transfected cells. This resulted in complete disappearance of high-molecular-weight HA species (Figure 3A-d). These findings suggest that CD44-mediated HA capture at the cell surface may be a key factor in enabling efficient HA degradation by TMEM2. The stronger effect observed when CD44 is co-expressed with TMEM2 further suggests that TMEM2-mediated HA degradation is most efficient when HA is retained on the same cell surface that expresses TMEM2.

We next asked whether the co-expression of CD44 with TMEM2 in the same cells could promotes degradation freely diffusible HA. FAHA-H2 was added to culture supernatants of 293T cells, and its degradation was analyzed by size-exclusion chromatography (Figure 3B). Mock-transfected cells and cells transfected with CD44 or TMEM2 alone showed little degradation of FA-HA (Figures 3B-a–c). In contrast, cells co-expressing TMEM2 and CD44 exhibited a marked reduction in high-molecular-weight HA species and a corresponding increase in partially depolymerized HA species (Figure 3B-d). These results indicate that the promoting effect of CD44 on TMEM2-mediated HA degradation is not limited to cell–cell contexts but also extends to freely diffusible HA. Taken together, these findings suggest that CD44-mediated HA capture or retention contributes to efficient TMEM2-mediated degradation under physiological conditions.

### 3.4 Link module–containing transmembrane proteins promote TMEM2-mediated hyaluronan degradation

**FIGURE 4:**
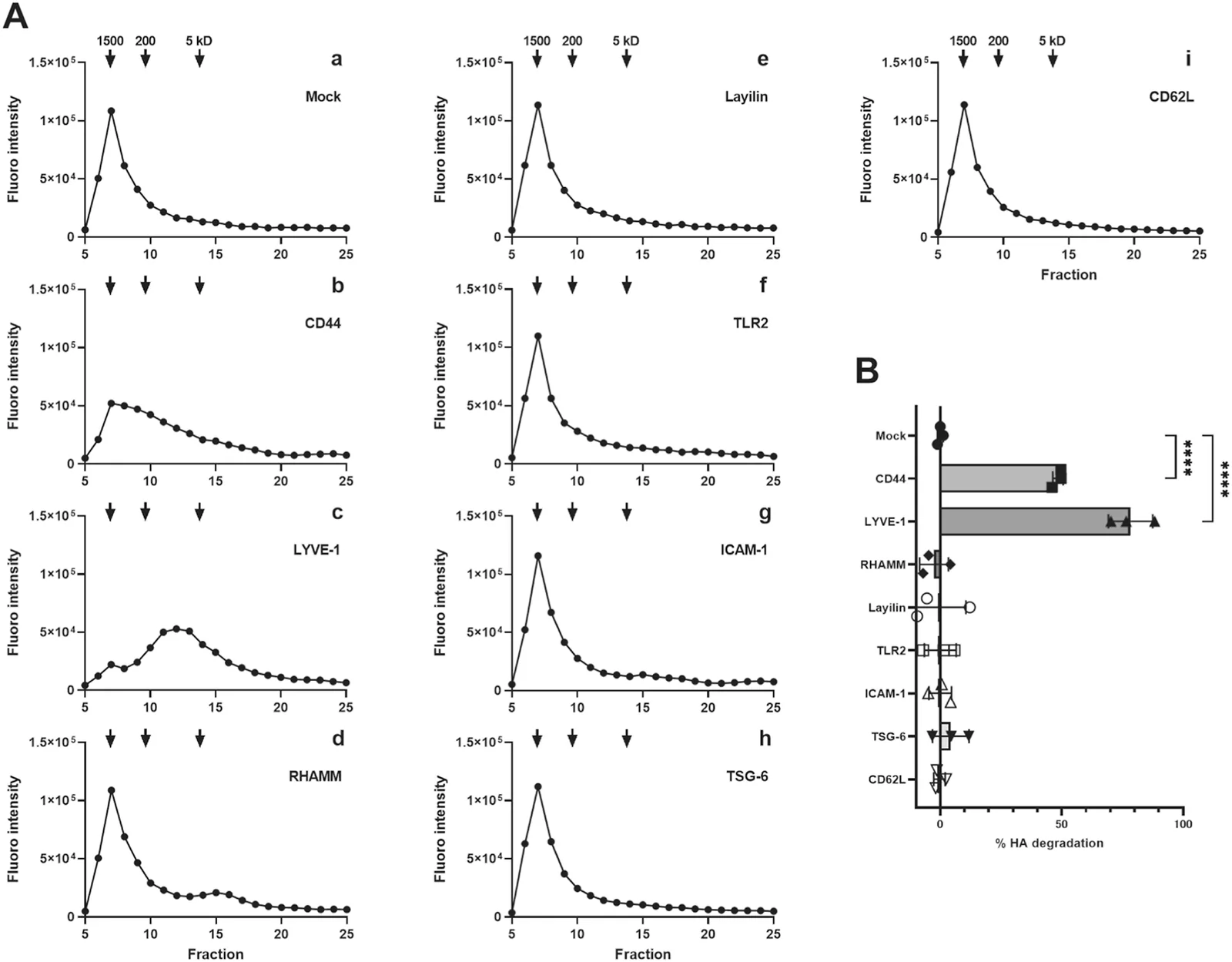
Link module–containing transmembrane proteins, but not other HA-binding proteins, promote TMEM2-mediated HA degradation. **(A)** FA-HA was incubated with 293^TMEM2^ cells transfected with the following HA-binding proteins: (a) none; (b) CD44; (c) LYVE-1; (d) RHAMM; (e) layilin; (f) TLR2; (g) TSG-6; (h) ICAM-1; and (i) CD62L. HA size distribution was analyzed by size-exclusion chromatography. **(B)** HA degradation activity was calculated from the reduction in cumulative signal intensity across fractions 5–8 relative to the untreated control. Data are presented as mean ± SD from three independent experiments. Statistical significance was determined using one-way ANOVA followed by Sidak’s multiple comparisons test. Data are presented as mean ± SD from three independent experiments. ****P < 0.0001; ns, not significant.

We next examined whether other HA-binding cell surface proteins promote TMEM2-mediated HA degradation. These include the CD44 paralogue LYVE-1 (Banerji et al., 1999), the receptor for hyaluronan-mediated motility (RHAMM; also known CD168) (Hardwick et al., 1992), the C-type lectin-like protein layilin (Bono et al., 2001), Toll-like receptor TLR2 (Jiang et al., 2007), and ICAM-1 (McCourt et al., 1994). CD44 and LYVE-1 are link module-containing transmembrane proteins, while other proteins are thought to bind HA via non-link module mechanisms. In these assays, we also included the soluble link module–containing HA-binding protein TSG-6 (Lee et al., 1992) and CD62L (L-selectin), which is not known to bind HA. We tested the effects of expressing each protein in *trans* assays using 293^TMEM2^ cells as the host (Figures 4A, a–i). Expression of transiently transfected TLR2, ICAM-1, and CD62L in 293^TMEM2^ cells was verified by flow cytometry, whereas expression of LYVE-1, RHAMM, layilin, and TSG-6 was verified by immunoblotting (Supplementary Figure S1). Among these proteins, only CD44 and LYVE-1 promoted TMEM2-mediated HA degradation (Figures 4A-b,c,B). Although protein expression levels were not quantitatively normalized across the different HA-binding proteins examined, their effects on HA-degradation activity differed strikingly with CD44 and LYVE-1 showing striking activity whereas the other proteins showed little or no activity. This finding strongly suggests the functional specificity of these link module–containing HA receptors in regulating TMEM2-mediated HA degradation in a cellular context.

## 4 Discussion

Our study identifies HA capture or retention at the cell surface as a key factor facilitating the hyaluronidase activity of human TMEM2. TMEM2 efficiently degrades endogenously synthesized HA when expressed in HA-producing cells but not when expressed in separate cells. Co-expression of TMEM2 with CD44 or LYVE-1 results in a marked enhancement of HA-degrading activity, whereas other known HA-binding proteins, including RHAMM, layilin, TLR2, ICAM-1, and TSG-6, do not exert this effect. These findings suggest that efficient TMEM2-mediated HA degradation is particularly favored when HA is retained at the same cell surface that expresses TMEM2, and that specific HA-binding receptors such as CD44 and LYVE-1 can promote this process.

### 4.1 Substrate confinement enhances TMEM2-mediated HA degradation

The *cis* requirement observed when TMEM2 was co-expressed with HAS3 suggests that a short-range spatial relationship between TMEM2 and its HA substrate is important for robust TMEM2-mediated HA degradation in cellular contexts. We speculate that cell surface retention of HA—achieved either by HAS-mediated tethering (Tammi et al., 1998) or by through interactions with CD44 or LYVE-1—helps maintain HA within a spatial range permissive for efficient TMEM2-mediated degradation. Such retention may increase the local substrate concentration near TMEM2 and prolong HA dwell time within its effective range, thereby facilitating processive cleavage by increasing the frequency and duration of TMEM2–substrate interactions. This framework may also explain the weak activity of TMEM2 toward freely diffusible HA observed in conventional cell-based assays (Sato et al., 2023). In the absence of substrate capture, TMEM2 encounters HA too infrequently or transiently to support measurable degradation, highlighting the importance of local HA organization for efficient TMEM2 activity.

### 4.2 Possible mechanistic basis for selective promotion of TMEM2-mediated hyaluronan degradation by CD44 and LYVE-1

Among the known HA-binding proteins tested, only CD44 and LYVE-1 enhanced TMEM2-dependent degradation in our system. This selectivity may be explained by the biochemical and structural properties of these receptors.

CD44 is a multivalent, high-avidity HA receptor whose binding strength increases with HA chain length owing to multivalent engagement and receptor density. Classical biochemical studies have demonstrated that CD44 exhibits monovalent binding to short HA oligomers but undergoes a sharp increase in avidity for longer HA chains (20–38 sugars), reflecting a transition to multivalent, high-affinity capture (Lesley et al., 2000). CD44 also undergoes homodimerization and nanoclustering. These features provide a plausible mechanism for concentrated HA capture at the nanoscale (Ma et al., 2022), which could facilitate TMEM2 access to spatially retained HA.

LYVE-1 exhibits a unique HA-binding architecture distinct from that of CD44, involving a “sliding” mechanism in which HA chain ends are drawn into a deep binding cleft through conformational rearrangements (Bano et al., 2025). This binding geometry may position HA in a configuration more permissive for TMEM2-mediated cleavage than the binding modes of the other HA-binding proteins, including CD44 (Bano et al., 2025), consistent with our observation that LYVE-1 promotes TMEM2-mediated HA degradation more strongly than CD44.

Moreover, these two link module-containing proteins not only bind HA but also modulate its organization at the cell surface. CD44 assembles HA into a dense (400–500 nm) pericellular glycocalyx, which serves as a ligand for LYVE-1 (Johnson et al., 2021). Such cooperative HA structuring supports the concept that these receptors generate spatially confined HA platforms at the cell surface that facilitate efficient TMEM2-mediated degradation. The lack of promoting activity by TSG-6 further suggests that HA binding or retention at the cell surface, rather than soluble HA binding alone, is important for supporting TMEM2-mediated degradation.

We also found that the other HA-binding proteins tested (RHAMM, layilin, TLR2, and ICAM-1) did not enhance HA degradation. Although these proteins have been reported to bind HA, their binding affinities, specificities, and functional significance remain less well characterized than those of CD44 and LYVE-1. The lack of effect in our system suggests that either their HA interactions are not sufficiently strong or that HA binding alone, without modulation of HA organization, is insufficient to support TMEM2 activity. It is possible that the mode of HA binding—such as multivalent capture, chain-end orientation, and nanoscale clustering—is the key determinant of whether an HA-binding protein can potentiate TMEM2.

### 4.3 Concluding remarks

Our study uncovers a novel mechanism regulating TMEM2 activity in which efficient HA degradation is favored by the local spatial relationship between TMEM2 and its substrate. Proximity of TMEM2 to sites of HA synthesis and/or its co-presence with CD44 or LYVE-1 may be a key factor enabling efficient TMEM2-mediated HA degradation. These findings help explain why this enzyme, despite its relatively modest catalytic activity *in vitro*, can exert profound effects on HA catabolism *in vivo*. Our findings also provide new insight into the regulation of the glycocalyx, which has recently attracted increasing attention for its roles in cell signaling, mechanotransduction, and matrix organization. HA is a major component of the glycocalyx, and CD44 has been implicated in its organization (Freeman et al., 2018). TMEM2 may therefore contribute to glycocalyx homeostasis by regulating cell-surface HA turnover in cooperation with CD44.

## Data Availability

The original contributions presented in the study are included in the article/Supplementary Material, further inquiries can be directed to the corresponding author.

## Supplementary material

SUPPLEMENTARY FIGURE 1 Confirmation of expression of transfected HA-binding proteins. Because reliable flow-cytometry-compatible antibodies were not available for all proteins examined, expression of the transfected constructs was verified by either flow cytometry or immunoblotting, depending on antibody compatibility. **(A)** Flow cytometric analysis of 293^TMEM2^ cells transfected with CD44, TLR2, ICAM-1, and CD62L. Overlaid histograms show the fluorescence intensity of mock-transfected control cells (*blue histogram*) and cells transfected with the indicated expression plasmids (*red histogram*), both stained with the same corresponding antibodies. **(B)** Immunoblotting analysis of 293^TMEM2^ cell transfected with LYVE-1, RHAMM, layilin, or TSG-6. β-actin was used as a loading control.
